# Allergenic food introduction and risk of childhood atopic diseases

**DOI:** 10.1371/journal.pone.0187999

**Published:** 2017-11-27

**Authors:** Niels J. Elbert, Jessica C. Kiefte-de Jong, Trudy Voortman, Tamar E. C. Nijsten, Nicolette W. de Jong, Vincent W. V. Jaddoe, Johan C. de Jongste, Roy Gerth van Wijk, Liesbeth Duijts, Suzanne G. M. A. Pasmans

**Affiliations:** 1 The Generation R Study Group, Erasmus MC, University Medical Center Rotterdam, Rotterdam, The Netherlands; 2 Department of Dermatology, Erasmus MC, University Medical Center Rotterdam, Rotterdam, The Netherlands; 3 Department of Epidemiology, Erasmus MC, University Medical Center Rotterdam, Rotterdam, The Netherlands; 4 Department of Pediatrics, Erasmus MC, University Medical Center Rotterdam, Rotterdam, The Netherlands; 5 Department of Global Public Health, Leiden University College, The Hague, The Netherlands; 6 Department of Internal Medicine, Division of Allergology, Erasmus MC, University Medical Center Rotterdam, Rotterdam, The Netherlands; 7 Department of Pediatrics, Division of Respiratory Medicine and Allergology, Erasmus MC, University Medical Center Rotterdam, Rotterdam, The Netherlands; 8 Department of Pediatrics, Division of Neonatology, Erasmus MC, University Medical Center Rotterdam, Rotterdam, The Netherlands; Université Paris Descartes, FRANCE

## Abstract

**Background:**

The role of timing and diversity of allergenic food introduction in the development of childhood allergic sensitization and atopic diseases is controversial.

**Objective:**

To examine whether timing and diversity of allergenic food introduction are associated with allergic sensitization, allergy and eczema in children until age 10 years.

**Materials and methods:**

This study among 5,202 children was performed in a population-based prospective cohort. Timing (age ≤6 months vs. >6 months) and diversity (0, 1, 2 and ≥3 foods) of allergenic food (cow's milk, hen's egg, peanut, tree nuts, soy and gluten) introduction were assessed by questionnaires at ages 6 and 12 months. At age 10 years, inhalant and food allergic sensitization were measured by skin prick tests, and physician-diagnosed inhalant and food allergy by questionnaire. Data on parental-reported physician-diagnosed eczema were obtained from birth until age 10 years.

**Results:**

Children introduced to gluten at age ≤6 months had a decreased risk of eczema (aOR (95% CI): 0.84 (0.72, 0.99)), compared with children introduced to gluten at age >6 months. However, timing of allergenic food introduction was not associated with allergic sensitization or physician-diagnosed allergy. Children introduced to ≥3 allergenic foods at age ≤6 months had a decreased risk of physician-diagnosed inhalant allergy (0.64 (0.42, 0.98)), compared with children not introduced to any allergenic food at age ≤6 months. However, diversity of allergenic food introduction was not associated with allergic sensitization, physician-diagnosed food allergy or eczema.

**Conclusion:**

Neither timing nor diversity of allergenic food introduction was consistently associated with childhood allergic sensitization, allergy or eczema.

## Introduction

The role of timing and diversity of allergenic food introduction in the development of childhood allergic sensitization and atopic diseases, such as allergy and eczema, is controversial. Currently, the World Health Organization and the American Academy of Pediatrics recommend not to introduce any complementary foods until age 6 months[1, 2], while the European Academy of Allergy and Clinical Immunology and the European Society for Pediatric Gastroenterology, Hepatology and Nutrition recommend not to avoid or delay the introduction of potentially allergenic foods beyond age 4 months, irrespective of atopic heredity[3, 4].

We previously demonstrated that the introduction of allergenic foods, such as cow's milk and peanut, at age ≤6 months was not associated with eczema until age 4 years[5]. Recently, the LEAP and LEAP-On trials showed that peanut introduction in high-risk children aged 4–11 months was associated with a decreased frequency of peanut allergy at age 5 years that persisted after 12 months of peanut avoidance[6, 7]. This resulted in addendum guidelines for the prevention of peanut allergy in high-risk children, recommending introduction of peanut as early as age 4–6 months in children with severe eczema, egg allergy or both[8]. Another trial did not show an effect of early introduction of six common allergenic foods on the frequency of food allergies between age 1 and 3 years among a selected group of exclusively breast-fed children from the general population[9]. However, less is known about the effects of the timing of introduction of common allergenic foods in early life on allergic sensitization, allergy and eczema in an unselected group of school-age children[10]. Previous birth cohort studies have examined the association of diversity of solid food introduction, by means of the number of solid foods introduced, with allergic sensitization and diseases,[11–15] but none of these studies focused specifically on allergenic foods.

Because animal studies suggest that acquiring immune tolerance is an active process and that exposure to dietary factors during a critical early window at age 4–6 months may be essential to this process[16], we hypothesized that timing and diversity of allergenic food introduction might induce immune tolerance and, subsequently, influence the risk of developing allergic sensitization and atopic diseases in childhood. Therefore, we aimed to examine among 5,202 children participating in a population-based prospective cohort study whether timing and diversity of introduction of allergenic foods (cow's milk, hen's egg, peanut, tree nuts, soy and gluten) were associated with the development of allergic sensitization, inhalant or food allergy and eczema until age 10 years.

## Materials and methods

### General design

This study was embedded in the Generation R Study, a population-based prospective cohort study from fetal life onwards[17]. The study has been approved by the Medical Ethical Committee of the Erasmus Medical Center, Rotterdam, The Netherlands (MEC-2012-165). Written informed consent was obtained from both parents or legal representatives. A total of 5,202 children were included for the current analyses (S1 Fig).

### Allergenic food introduction

We collected data on the introduction of cow's milk, hen's egg, peanut, tree nuts, soy and gluten by parental questionnaires at ages 6 and 12 months ("How often do you give your child cow's milk/hen's egg/peanuts/tree nuts/soy/gluten at present?" and "How old was your child when you first gave it cow's milk/hen's egg/peanuts/tree nuts/soy/gluten?"). Data from the questionnaires were combined and categorized into 'introduction at age ≤6 months' and 'introduction at age >6 months'[5]. In case of inconsistencies between data collected at ages 6 and 12 months, we considered the data collected at age 6 months as the reference, since weaning generally starts around that age[18]. Furthermore, reported introductions of specific allergenic foods were cross-checked with a short food-frequency questionnaire also completed by the mother when the child was 6 and 12 months old.[19] This questionnaire consisted of food products frequently consumed by children around these ages according to a Dutch food consumption survey[18]. For example, if parents indicated that they had never introduced peanut in the child's diet at age 12 months, but at age 6 months parents reported that the child had consumed peanut butter more than once, then the introduction of peanut was considered to be at age ≤6 months. Additionally, the introduction of cow's milk and soy was cross-checked with the type of bottle feeding (regular, soy-based or based on fully or partly hydrolyzed whey protein), and the introduction of gluten was cross-checked with the consumption of specific brands of bread, biscuits and porridge (gluten-containing or gluten-free brands) at ages 6 and 12 months. To assess diversity of allergenic food introduction at age ≤6 months, we categorized the number of allergenic foods introduced at this age into '0', '1', '2' and '≥3'.

### Allergic sensitization, allergy and eczema

Children visited the research center at a median age of 9.7 years (2.5–97.5th percentile: 9.3–10.5). Inhalant and food allergic sensitization to house dust mite, 5-grass mixture, birch, cat and dog (ALK-Abelló B.V., Almere, The Netherlands), and hazelnut, cashew nut, peanut and peach were measured by skin prick tests using the scanned area method[20]. Questions adapted from the International Study of Asthma and Allergies in Childhood core questionnaires provided information on physician-diagnosed inhalant ("Was your child ever diagnosed with an allergy to pollen (hay fever)/house dust mite/cat/dog?") (no; yes) and food ("Was your child ever diagnosed with an allergy to cashew nut/peanut?") (no; yes) allergy at age 10 years[21]. We further combined allergic sensitization and physician-diagnosed allergy into groups of 'no allergic sensitization and no allergy', 'any allergic sensitization, but no allergy', 'no allergic sensitization, but any allergy', and 'any allergic sensitization and any allergy'. Physician-diagnosed eczema was parental-reported at ages 6 months and 1, 2, 3, 4 and 10 years ("Was your child diagnosed with eczema in the last 6 months/last year?") (no; yes).

### Covariates

Information on maternal age, education (primary or secondary; higher), history of allergy, eczema or asthma (no; yes), parity (nulliparous; multiparous), pet keeping (no; yes) and body mass index (BMI) was obtained by questionnaires completed by the mother at enrollment. Information on maternal smoking (no; yes) was obtained by postal questionnaires multiple times during pregnancy. We assessed maternal psychiatric symptoms in the second trimester of pregnancy using the Global Severity Index of the Brief Symptom Inventory[22]. Information on child's sex, gestational age at birth and birth weight was obtained from obstetric and midwife records at birth. We based ethnic origin (European; non-European) of the child on the country of birth of the parents[23]. Delivery reports and postal questionnaires completed by the mother when the child was 2, 6 and 12 months old provided data on ever breastfeeding (no; yes) and breastfeeding duration (never; <6 months; ≥6 months). We obtained information on ointment use for eczema (no; yes), physician-diagnosed cow's milk allergy (no; yes), day care attendance (no; yes) and antibiotic use (no; yes) by questionnaires at ages 2, 6 and 12 months. BMI was calculated from the child's weight and height measured at age 10–13 months during a visit to the research center.

### Statistical analysis

We used logistic regression or multinomial logistic regression models to examine the associations of timing and diversity of allergenic food introduction with the risk of allergic sensitization and physician-diagnosed allergy or combined allergic sensitization and allergy groups, respectively, at age 10 years. We used generalized estimating equation models to examine the associations of timing and diversity of allergenic food introduction with the longitudinal odds of eczema at ages 6 months and 1, 2, 3, 4 and 10 years independently and overall, taking into account correlations between repeated measurements of eczema within the same child[24]. First, we adjusted for potential confounders, including maternal age at enrollment, education, history of allergy, eczema or asthma, parity, pet keeping, BMI at enrollment, smoking, psychiatric symptoms, and child's sex, gestational age, birth weight, ethnic origin, breastfeeding, day care attendance and antibiotic use. We considered this the main model. Second, child's BMI at age 10–13 months was considered as an intermediate and additionally adjusted for in the model. Confounders were included in the models based on literature, if they were associated with both the determinant and the outcome, or if they changed the effect estimates with ≥10%. Analyses with inhalant or food allergic sensitization or allergy as the outcomes were mutually adjusted for each other. Tests for trends were performed by including diversity of allergenic food introduction as a continuous variable in the models. Because the associations evaluated in the current study are complementary and share a single underlying biological hypothesis, we did not apply multiple testing correction[25]. We performed additional analyses to assess the robustness of our results, accounting for disease-related modification of the exposure and effect modification. First, we performed risk period-specific sensitivity analyses by excluding children who developed eczema until age 6 months (n = 928). Second, we additionally adjusted our main model for ointment use for eczema at age 2 months. Third, we tested the modifying effects of maternal history of allergy, eczema or asthma, and child's breastfeeding duration and history of cow's milk allergy until age 1 year, and the time-varying effect of age at eczema measurement by adding them as product terms with the allergenic food variables in the models. Missing data of covariates, allergenic foods and eczema were multiple-imputed to reduce potential bias associated with missing data (S1 Table). The best indicator for the presence or absence of eczema is an eczema measurement at a different age. Therefore, at least one eczema measurement was available in our population for analysis to predict other eczema measurements correctly. Because we lacked repeated measurements on allergic sensitization and physician-diagnosed allergy, we did not impute missing data of these outcomes. The size or direction of the effect estimates did not materially differ between analyses with imputed data and complete cases only (data not shown). Therefore, we present pooled results based on imputed analyses only. Measures of association are presented as adjusted odds ratios (aOR) with their 95% confidence intervals (CI). Statistical analyses were performed using SPSS 21.0.0.1 for Windows (IBM Corp., Armonk, NY, USA) and SAS 9.4 (SAS Institute Inc., Cary, NC, USA).

## Results

### General

The majority of children were introduced to at least 1 allergenic food (81.8%; n = 4,257) at age ≤6 months, most commonly cow's milk (74.0%; n = 3,847) and gluten (55.8%; 2,904) (Table 1 and S2 Table). Inhalant or food allergic sensitization was present in 31.5% (n = 949) and 6.7% (n = 202) of the children at age 10 years, respectively. Physician-diagnosed inhalant or food allergy was present in 12.0% (n = 435) and 2.3% (n = 81) of the children at age 10 years, respectively. The prevalence of eczema declined from 17.8% (n = 928) at age 6 months to 8.6% (n = 448) at age 10 years. Mothers without follow-up data were younger, lower educated, had higher parity, more often kept pets during pregnancy, had a higher BMI at enrollment, and smoked more and had more psychiatric symptoms during pregnancy. Their children were more often males, born younger, had a lower birth weight, and were more often of non-European origin and less often breastfed and exposed to gluten at age ≤6 months (S3 Table).

**Table 1:** Characteristics of mothers and their children.

	n = 5,202
**Maternal characteristics**	
Age at enrollment (years)*	31.1 (4.8)
Education, higher (%)	53.5 (2,784)
History of allergy, eczema or asthma, yes (%)	37.9 (1,972)
Parity, ≥1 (%)	41.5 (2,158)
Pet keeping during pregnancy, yes (%)	34.9 (1,814)
Body mass index at enrollment (kg/m^2^)^†^	23.6 (18.8–35.6)
Smoking during pregnancy, yes (%)	23.0 (1,195)
Psychiatric symptoms during pregnancy^†^	0.13 (0–1.33)
**Child characteristics**	
Sex, female (%)	50.4 (2,623)
Gestational age at birth (weeks)^†^	40.1 (36.0–42.3)
Birth weight (grams)*	3,454 (549)
Ethnic origin, European (%)	72.5 (3,770)
Breastfeeding (%)	
Ever, yes	91.6 (4,763)
Duration, ≥6 months	34.9 (1,814)
Ointment use for eczema at age 2 months, yes (%)	7.6 (393)
Cow's milk allergy until age 1 year, yes (%)	5.9 (305)
Day care attendance until age 1 year, yes (%)	57.5 (2,992)
Antibiotic use until age 1 year, yes (%)	22.0 (1,143)
Body mass index at age 10–13 months (kg/m^2^)^†^	17.3 (14.9–20.3)
Timing of allergenic food introduction, ≤6 months (%)	
Cow's milk	74.0 (3,847)
Hen's egg	14.2 (741)
Peanut	5.8 (303)
Tree nuts	4.5 (236)
Soy	20.3 (1,055)
Gluten	55.8 (2,904)
Diversity of allergenic foods introduced at age ≤6 months (%)	
No allergenic foods introduced	18.2 (945)
1 allergenic food introduced	33.7 (1,754)
2 allergenic foods introduced	29.1 (1,516)
≥3 allergenic foods introduced	19.0 (987)
Allergic sensitization at age 10 years, yes (%)	
Inhalant	31.5 (949)
Food	6.7 (202)
Physician-diagnosed allergy at age 10 years, yes (%)	
Inhalant	12.0 (435)
Food	2.3 (81)
Allergic sensitization and allergy combined at age 10 years (%)	
No allergic sensitization and no allergy	67.1 (1,759)
Any allergic sensitization, but no allergy	21.9 (574)
No allergic sensitization, but any allergy	1.2 (31)
Any allergic sensitization and any allergy	9.8 (258)
Eczema, yes (%)	
Age 6 months	17.8 (928)
Age 1 year	13.3 (690)
Age 2 years	13.7 (712)
Age 3 years	10.0 (519)
Age 4 years	8.5 (440)
Age 10 years	8.6 (448)

Values are *means (SD), ^†^medians (2.5–97.5th percentile) or percentages (absolute numbers) based on imputed data. Data on allergic sensitizations and physician-diagnosed allergies are not imputed.

### Allergenic food introduction

**Fig 1:**
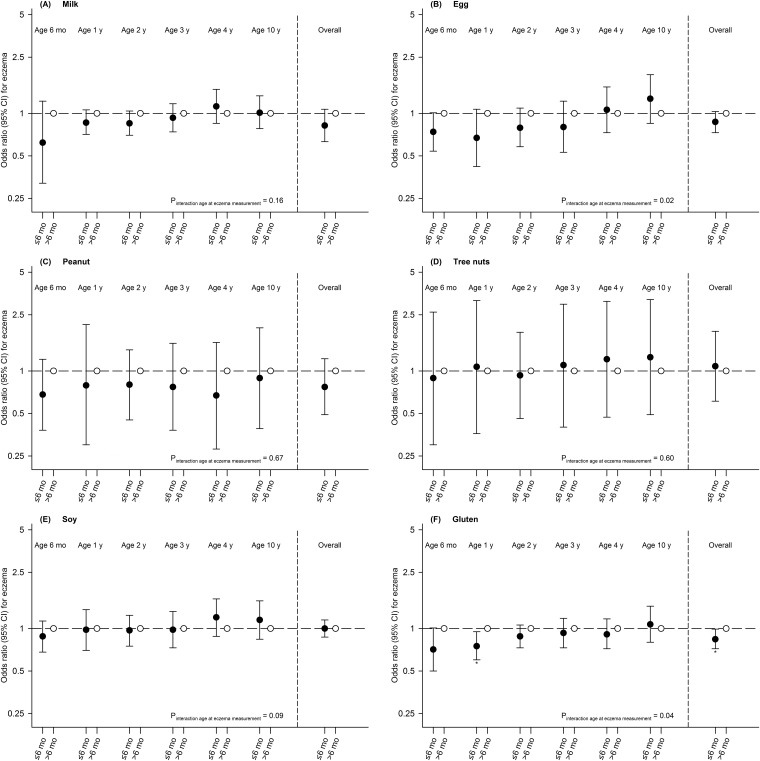
Associations of timing of milk (A), egg (B), peanut (C), tree nuts (D), soy (E) and gluten (F) introduction with eczema in children until age 10 years. Values are odds ratios (95% confidence interval) from generalized estimating equation models based on imputed data. Reference group is children with allergenic food introduction at age >6 months. Models are adjusted for maternal age at enrollment, education, history of allergy, eczema or asthma, parity, pet keeping, body mass index at enrollment, smoking, psychiatric symptoms, and child's sex, gestational age, birth weight, ethnic origin, breastfeeding, day care attendance and antibiotic use. *P-value <0.05. Mo = months; y = year(s).

**Fig 2:**
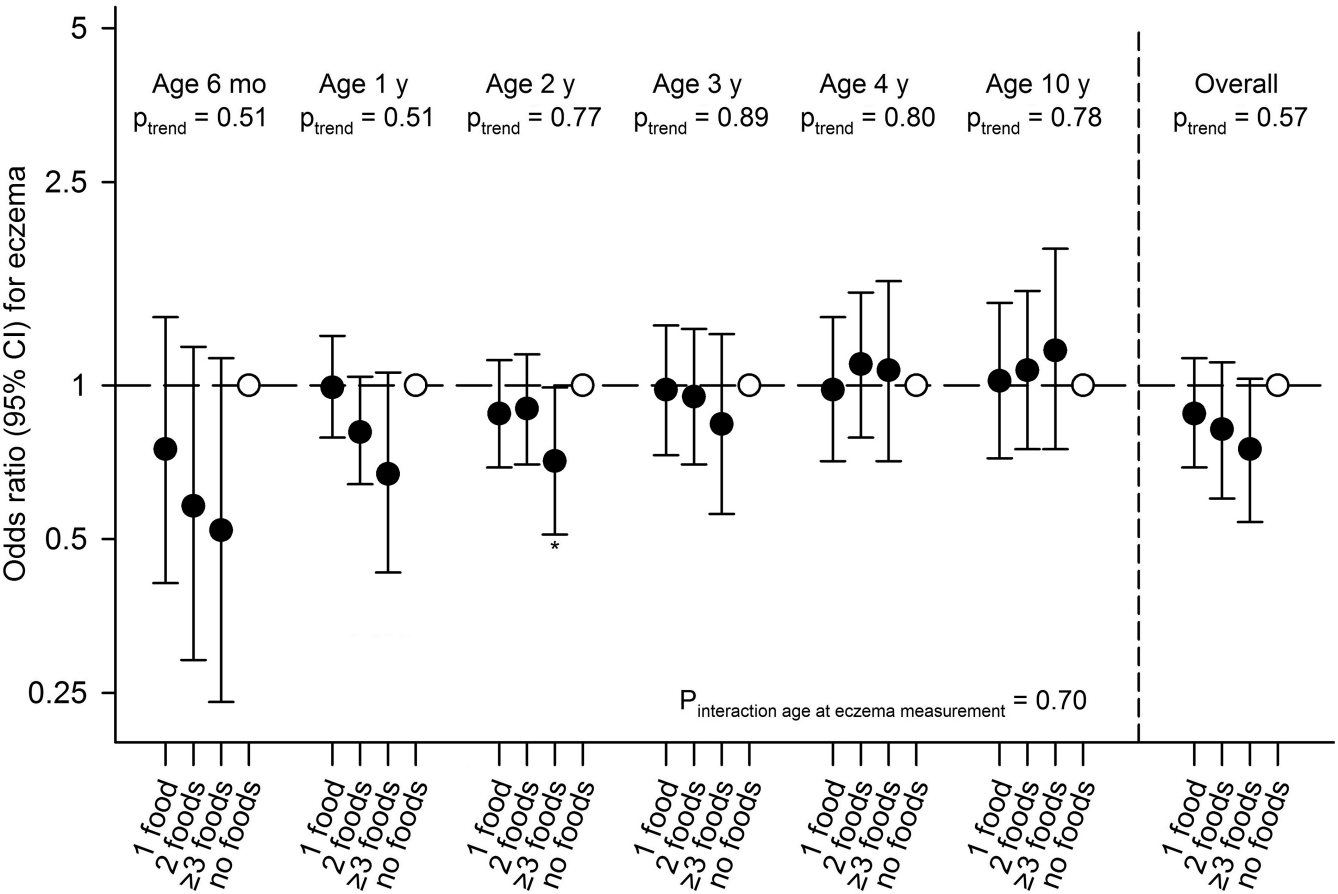
Associations of diversity of allergenic food introduction with eczema in children until age 10 years. Values are odds ratios (95% confidence interval) from generalized estimating equation models based on imputed data. Reference group is children with no allergenic foods introduced at age ≤6 months. Models are adjusted for maternal age at enrollment, education, history of allergy, eczema or asthma, parity, pet keeping, body mass index at enrollment, smoking, psychiatric symptoms, and child's sex, gestational age, birth weight, ethnic origin, breastfeeding, day care attendance and antibiotic use. *P-value <0.05. Mo = months; y = year(s).

Timing of allergenic food introduction was not associated with allergic sensitization, physician-diagnosed allergy or combined allergic sensitization and allergy groups (Table 2 and S4 Table). Children introduced to gluten at age ≤6 months had a decreased risk of eczema until age 10 years (aOR (95% CI): 0.84 (0.72, 0.99)), compared with children introduced to gluten at age >6 months (Fig 1 and S5 Table). Timing of introduction of other allergenic foods was not associated with eczema at specific ages or overall. Diversity of allergenic food introduction was not associated with allergic sensitization, physician-diagnosed food allergy or combined allergic sensitization and allergy groups (Table 2 and S4 Table). Children introduced to ≥3 allergenic foods at age ≤6 months had a decreased risk of physician-diagnosed inhalant, but not food allergy at age 10 years (0.64 (0.42, 0.98)), compared with children not introduced to any allergenic food at age ≤6 months (Table 2). We did not observe a significant trend for a lower risk of physician-diagnosed inhalant allergy when introduced to a higher number of allergenic foods at age ≤6 months (p = 0.05). Diversity of allergenic food introduction was not consistently associated with eczema (Fig 2 and S5 Table). Additional adjustment for child's BMI at age 10–13 month did not materially affect the size and the direction of the effect estimates (data not shown).

**Table 2:** Associations of timing and diversity of allergenic food introduction with allergic sensitizations and physician-diagnosed allergies in children at age 10 years.

	Odds ratio (95% confidence interval) for allergic sensitization		Odds ratio (95% confidence interval) for physician-diagnosed allergy	
	Inhalant n = 3,017	Food n = 3,006	Inhalant n = 3,617	Food n = 3,546
Allergenic food introduced at age ≤6 months*				
Cow's milk (n = 3,847)	0.99 (0.81, 1.22)	0.76 (0.50, 1.17)	0.83 (0.65, 1.08)	1.44 (0.76, 2.73)
Hen's egg (n = 741)	0.99 (0.76, 1.31)	1.06 (0.61, 1.85)	0.74 (0.48, 1.15)	0.83 (0.32, 2.13)
Peanut (n = 303)	0.74 (0.46, 1.20)	1.68 (0.69, 4.10)	0.59 (0.26, 1.37)	2.55 (0.67, 9.67)
Tree nuts (n = 236)	1.07 (0.55, 2.09)	0.63 (0.09, 4.34)	0.84 (0.26, 2.71)	2.43 (0.47, 12.45)
Soy (n = 1,055)	1.05 (0.85, 1.30)	0.91 (0.57, 1.45)	0.97 (0.72, 1.32)	1.39 (0.72, 2.68)
Gluten (n = 2,904)	0.99 (0.83, 1.19)	0.71 (0.48, 1.06)	0.80 (0.63, 1.02)	0.65 (0.36, 1.18)
Diversity of allergenic foods introduced at age ≤6 months^†^				
1 allergenic food introduced (n = 1,754)	0.97 (0.75, 1.25)	0.90 (0.53, 1.53)	0.86 (0.63, 1.19)	1.56 (0.73, 3.32)
2 allergenic foods introduced (n = 1,516)	0.94 (0.72, 1.22)	0.76 (0.44, 1.32)	0.85 (0.61, 1.18)	0.77 (0.33, 1.82)
≥3 allergenic foods introduced (n = 987)	1.01 (0.75, 1.36)	0.69 (0.36, 1.31)	**0.64 (0.42, 0.98)**	1.56 (0.63, 3.86)
P-value for trend	0.96	0.18	0.05	0.92

Values are odds ratios (95% confidence interval) from logistic regression models based on imputed data. Bold values indicate statistical significance at the α = 0.05 level. Reference group is children with *allergenic food introduction at age >6 months or ^†^no allergenic foods introduced at age ≤6 months. Models are adjusted for maternal age at enrollment, education, history of allergy, eczema or asthma, parity, pet keeping, body mass index at enrollment, smoking, psychiatric symptoms, and child's sex, gestational age, birth weight, ethnic origin, breastfeeding, day care attendance and antibiotic use, and mutually for inhalant and food allergic sensitization or allergy.

### Additional analyses

Risk period-specific sensitivity analyses showed that effect estimates for the associations of early gluten introduction with eczema until age 10 years, and of introduction of ≥3 allergenic foods with physician-diagnosed inhalant allergy attenuated to non-significant (0.95 (0.81, 1.10) and 0.65 (0.39, 1.08), respectively). Effect estimates did not materially change in size or direction when we additionally adjusted our main analyses for ointment use for eczema at age 2 months (data not shown). Results were similar among children with and without a maternal history of allergy, eczema or asthma or a history of cow's milk allergy until age 1 year, and we observed no modifying effect of child's breastfeeding duration on any of the examined associations (p-values for interaction >0.05). For egg and gluten introduction, a time-varying effect of age at eczema measurement was observed with stronger associations of egg and gluten introduction with eczema in early than in later life (p-values for interaction <0.05) (Fig 1).

## Discussion

In this large prospective population-based study, we observed no consistent association of timing and diversity of allergenic food introduction with allergic sensitization or atopic diseases in children until age 10 years. However, children introduced to gluten at age ≤6 months had a decreased risk of eczema until age 10 years, and children introduced to ≥3 allergenic foods at age ≤6 months had a decreased risk of physician-diagnosed inhalant allergy at age 10 years.

### Comparison of main findings with other studies

For the allergenic foods evaluated in the current study, a recent meta-analysis showed no association of early allergenic food introduction with inhalant or food allergic sensitization, measured by skin prick tests or antigen-specific immunoglobulin E (IgE), allergic rhinitis, and eczema[26]. However, introduction of hen's egg at age 4–6 months was associated with a 44% decreased risk of egg allergy at age 1 year, and introduction of peanut at age 4–11 months was associated with a 71% decreased risk of peanut allergy at age 3–5 years. There was no consistent evidence for an association of early cow's milk, tree nuts, soy or gluten introduction with food allergies. Previous birth cohort studies showed inconsistent results on the association of diversity of solid food introduction, by means of the number of solid foods introduced, with the risk of childhood allergic sensitization and atopic diseases[11–15]. However, literature focusing specifically on diversity of allergenic food introduction is lacking. Differences between results from the meta-analysis and the current study might be explained by differences in study population (general population vs. high-risk children; different age groups) and study aim (observational vs. tolerance induction for specific allergenic foods). Other possible explanations may include differences in recall bias, selection of allergenic foods, definitions of diversity of food introduction (solid vs. specific allergenic foods; number of foods per group), outcome methods (antigen-specific IgE vs. skin prick tests; physician-diagnosed vs. parental-reported), child's age at time of outcome measurement and measurement of and adjustment for potential confounders.

### Interpretation of results

Dietary factors may serve as substrates for the production of microbial metabolites that regulate immune activity and immune tolerance mechanisms[27]. Therefore, we hypothesized that early allergenic food introduction might influence immune tolerance and, subsequently, the development of childhood allergic sensitization and atopic diseases. However, we found no consistent association of the timing of introduction of allergenic foods with childhood allergic sensitization or atopic diseases. We did find an inverse association of early gluten introduction with eczema, which might be explained by the fact that when gluten are introduced to older children, the amounts tend to be greater than in younger children[28]. We can speculate that a higher gluten load may have resulted in T-cell activation rather than immune tolerance[28]. We observed an association of early gluten introduction with eczema overall but not consistently per year. This might be explained by increased statistical power when using eczema overall rather than a chance finding. Differences in observed associations of allergenic food introduction with eczema and allergic sensitizations or physician-diagnosed allergies might be due to differences in timing of these outcome measurements. Eczema was assessed longitudinally, while allergic sensitizations and physician-diagnosed allergies were measured at one time point only.

It is suggested that exposure to a variety of food products during the first year of life, especially beyond age 6 months, may be important for the development of immune tolerance[13, 14]. Again, we observed no consistent association of diversity of allergenic food introduction with allergic sensitization or atopic diseases. We did observe a suggestive trend for a lower risk of physician-diagnosed inhalant allergy when introduced to a higher (i.e., per 1-food group increase) number of allergenic foods. A recent birth cohort study showed that introduction of a higher number of solid foods was associated with a decreased expression of Cε germline transcript, a marker for antibody isotype switching of B-cells to IgE-producing cells[14]. The inhibition of isotype switching to IgE is one of the mechanisms that might be involved in the inhibition of atopic diseases by regulatory T cells[29]. We did not find an association of diversity of allergenic food introduction with food allergic sensitization and physician-diagnosed food allergy, which could partly be explained by the low prevalence of food allergic sensitization (6.7%) and physician-diagnosed food allergy (2.3%). Therefore, these results should be interpreted with caution.

Our findings might be explained by disease-related modification of the exposure, meaning that early symptoms of allergy or eczema in the child may encourage parents to alter feeding practices. Among children with early allergy-related symptoms and among those with a parental history of allergy, eczema or asthma, introduction of complementary foods, especially allergenic foods, tends to be delayed[14]. We tried to assess the effects of such bias by performing risk period-specific sensitivity analyses and additional adjustment for ointment use at age 2 months. We showed that to some degree disease-related modification of the exposure was present in our study, particularly for the association of early gluten introduction with eczema until age 10 years. Therefore, caution is warranted in interpreting our results, which require further studies for replication and exploration of underlying pathophysiological mechanisms. We found no modifying effects of maternal history of allergy, eczema or asthma and child’s breastfeeding duration and history of cow’s milk allergy until age 1 year.

### Strengths and limitations

The strengths of this study are the use of a population-based prospective study design from fetal life onwards with a large number of participants and detailed information on allergic sensitization, allergy and eczema. Also, we adjusted for multiple social, behavioral and environmental factors. However, some methodological limitations should be considered. First, characteristics of non-included subjects differed from those included in the study. Although this may affect the generalizability of our results, it is unlikely that these differences affected the observed associations. Second, our data did not allow us to evaluate the effects of allergenic food introduction during the specific window of immunological opportunity to induce immune tolerance at age 4–6 months. Instead, we assessed the associations of allergenic food introduction at age ≤6 months, which may have underestimated our results. Also, our data did not allow us to evaluate the effects of continued breastfeeding during the period of allergenic food introduction, which may be important for promoting immune tolerance[16]. However, we did not observe any modifying effect of breastfeeding duration. It has recently been suggested that the prevention of food allergy by means of early introduction of multiple allergenic foods is dose-dependent[9], but we lacked data on the precise amounts of allergenic foods introduced. Third, we cannot rule out that our results may be affected by disease-related modification of the exposure. Randomized controlled trials are required to completely exclude this potential bias[14]. Fourth, the short food-frequency questionnaire has previously been validated in our cohort for gluten introduction,[19] but not for the introduction of other allergenic foods. Non-differential misclassification of the timing of allergenic food introduction may have also occurred due to possible exposure of the child to 'hidden' allergenic foods,[30] which may have underestimated our results. To minimize the effect of 'hidden' allergenic food exposure, we performed cross-checks of the reported introductions of specific allergenic foods with a short food-frequency questionnaire consisting of food products, with the type of bottle feeding for the introduction of cow's milk and soy specifically, and with the consumption of specific brands of bread, biscuits and porridge for the introduction of gluten specifically. For measuring allergic sensitization, we selected a panel of common inhalant and food allergens relevant to children of age 10 years. Other allergens, such as milk and egg, were not considered because of low sensitization rates at this age[31]. The scanned area method is recommended in research settings and corrects for interobserver variability and ethnic differences in skin response to histamine[20]. Fifth, we did not perform double blind placebo-controlled food challenges or physical examinations to establish a diagnosis of food allergy or eczema, respectively. This may have led to non-differential misclassification of these outcomes and most probably an underestimation of the observed effects. Instead, we used parental questionnaires with widely accepted and commonly used questions that reliably reflect the prevalence of eczema in young children at the population level[21, 32]. Finally, as in any observational study, residual confounding due to insufficiently or unmeasured confounders might still be present.

In conclusion, we observed no consistent association of timing and diversity of allergenic food introduction with childhood allergic sensitization, physician-diagnosed allergy or eczema. Children introduced to gluten and those introduced to ≥3 allergenic foods at early age had a decreased risk of eczema or physician-diagnosed inhalant allergy, respectively. However, these results do not provide strong evidence to change current feeding guidelines. Further studies are needed to replicate our findings and to explore the specific underlying pathophysiological mechanisms.

## Supporting information

S1 Fig Flowchart of participants. (DOCX)

S1 Table Details of the multiple imputation model. *Also included in the multiple imputation model as indicator variables.
(DOCX)

S2 Table Characteristics of mothers and their children (n = 5,202). Values are *means (SD), ^†^medians (2.5–97.5th percentile) or percentages (absolute numbers) based on observed and imputed data. Data on allergic sensitizations and physician-diagnosed allergies are not imputed.
(DOCX)

S3 Table Characteristics of mothers and children included and not included in the study. Values are *means (SD), ^†^medians (2.5–97.5th percentile) or percentages (absolute numbers) based on observed data. P-values for difference are calculated by independent samples T-test for continuous variables with a normal distribution, the Mann-Whitney U test for continuous variables with a skewed distribution, and Pearson's Chi-square test for categorical variables. Bold values indicate statistical significance at the α = 0.05 level.
(DOCX)

S4 Table Associations of timing and diversity of allergenic food introduction with combined allergic sensitization and physician-diagnosed allergy groups in children at age 10 years. Values are odds ratios (95% confidence interval) from multinomial logistic regression models based on imputed data. Reference group is children without any allergic sensitization or physician-diagnosed allergy (n = 1,759), and with *allergenic food introduction at age >6 months or ^†^no allergenic foods introduced at age ≤6 months. Models are adjusted for maternal age at enrollment, education, history of allergy, eczema or asthma, parity, pet keeping, body mass index at enrollment, smoking, psychiatric symptoms, and child's sex, gestational age, birth weight, ethnic origin, breastfeeding, day care attendance and antibiotic use. N.A. = not available due to low number of children.
(DOCX)

S5 Table Associations of timing and diversity of allergenic food introduction with eczema per year and overall in children until age 10 years. Values are odds ratios (95% confidence interval) from generalized estimating equation models based on imputed data. Bold values indicate statistical significance at the α = 0.05 level. Reference group is children without any allergic sensitization or physician-diagnosed allergy, and with *allergenic food introduction at age >6 months or ^†^no allergenic foods introduced at age ≤6 months. Models are adjusted for maternal age at enrollment, education, history of allergy, eczema or asthma, parity, pet keeping, body mass index at enrollment, smoking, psychiatric symptoms, and child's sex, gestational age, birth weight, ethnic origin, breastfeeding, day care attendance and antibiotic use.
(DOCX)

## Abbreviations

aOR: adjusted odds ratio
BMI: body mass index
CI: confidence interval
IgE: immunoglobulin E
