# Supplementary material for: Allergenic food introduction and risk of childhood atopic diseases
Source: PLoS One. 2017 Nov 27;12(11):e0187999. doi: 10.1371/journal.pone.0187999 (PMC5703454; doi:10.1371/journal.pone.0187999)
Supplement: S1 Fig — (DOCX) [file pone.0187999.s001.docx]

**S1 Fig. Flowchart of participants.**

**n = 14**

Children without data on allergic sensitization, allergy or eczema excluded

**n = 3,228**

Twins (n = 208) and children without data on allergenic food introduction (n = 3,020) excluded

**n = 8,444**

Children with consent until phase 3 of the Generation R Study

**n = 5,216**

Children with data on allergenic food introduction available

**n = 5,202**

Children with any data on allergic sensitization, allergy or eczema available

Allergic sensitization at age 10 years

Inhalant n = 3,017

Food n = 3,006

Physician-diagnosed allergy at age 10 years

Inhalant n = 3,617

Food n = 3,546

Eczema

Age 6 months n = 3,776

Age 1 year n = 4,598

Age 2 years n = 4,382

Age 3 years n = 4,081

Age 4 years n = 4,012

Age 10 years n = 3,692
