# Supplementary material for: Allergenic food introduction and risk of childhood atopic diseases
Source: PLoS One. 2017 Nov 27;12(11):e0187999. doi: 10.1371/journal.pone.0187999 (PMC5703454; doi:10.1371/journal.pone.0187999)
Supplement: S1 Table — *Also included in the multiple imputation model as indicator variables. (DOCX) [file pone.0187999.s002.docx]

**S1 Table. Details of the multiple imputation model.**

| **Software used:** SPSS 21.0.0.1 for Windows (IBM Corp., Armonk, NY, USA) |
| --- |
| **Imputation method used:** fully conditional specification |
| **Model type for scale variables used:** predictive mean matching |
| **Number of imputed datasets created:** 25 |
| **Maximum number of iterations:** 20 |
| **Imputed variables*:** |
| *Outcomes:* eczema at ages 6 months and 1, 2, 3, 4 and 10 years |
| *Determinants:* introduction of milk, egg, peanut, tree nuts, soy and gluten |
| *Covariates:* maternal age, education, history of allergy, eczema or asthma, parity, pet keeping during pregnancy, body mass index at enrollment, smoking during pregnancy, and psychiatric symptoms during pregnancy; child's sex, gestational age at birth, birth weight, ethnic origin, breastfeeding ever, day care attendance until age 1 year, antibiotic use until age 1 year, body mass index at age 10-13 months, cow's milk allergy at age 1 year, and ointment use for eczema at age 2 months |
| **Additional indicator variables:** |
| *Outcomes:* ever eczema at age 10 years; histamine equivalent intracutaneous coefficient value for house dust mite, birch, 5-grass mixture, dog, cat, cashew, peanut and peach; physician-diagnosed allergy to pollen, house dust mite, cat, dog, peanut and cashew nut |
| *Determinants:* introduction of fruits or vegetables |
| *Covariates:* paternal age, education, history of allergy, eczema or asthma, body mass index at enrollment, smoking during pregnancy, and psychiatric symptoms during pregnancy; child's exclusiveness of breastfeeding; household income |
| **Treatment of binary or categorical variables:** logistic |
| **Statistical interactions included in imputation models:** none |

*Also included in the multiple imputation model as indicator variables.
