# Supplementary material for: Allergenic food introduction and risk of childhood atopic diseases
Source: PLoS One. 2017 Nov 27;12(11):e0187999. doi: 10.1371/journal.pone.0187999 (PMC5703454; doi:10.1371/journal.pone.0187999)
Supplement: S2 Table — Values are *means (SD), †medians (2.5–97.5th percentile) or percentages (absolute numbers) based on observed and imputed data. Data on allergic sensitizations and physician-diagnosed allergies are not imputed. (DOCX) [file pone.0187999.s003.docx]

**S2 Table. Characteristics of mothers and their children (n = 5,202).**

|  | **Observed** | **Imputed** |
| --- | --- | --- |
| **Maternal characteristics** |  |  |
| Age at enrollment (years)* | 31.1 (4.8) | 31.1 (4.8) |
| *Missing* | *0 (0)* | *0 (0)* |
| Education (%) |  |  |
| Primary or secondary | 44.8 (2,229) | 46.5 (2,418) |
| Higher | 55.2 (2,751) | 53.5 (2,784) |
| *Missing* | *4.3 (222)* | *0 (0)* |
| History of allergy, eczema or asthma (%) |  |  |
| No | 60.9 (2,658) | 62.1 (3,230) |
| Yes | 39.1 (1,705) | 37.9 (1,972) |
| *Missing* | *16.1 (839)* | *0 (0)* |
| Parity (%) |  |  |
| 0 | 58.7 (2,984) | 58.5 (3,044) |
| ≥1 | 41.3 (2,102) | 41.5 (2,158) |
| *Missing* | *2.2 (116)* | *0 (0)* |
| Pet keeping during pregnancy (%) |  |  |
| No | 65.4 (2,774) | 65.1 (3,388) |
| Yes | 34.6 (1,467) | 34.9 (1,814) |
| *Missing* | *18.5 (961)* | *0 (0)* |
| Body mass index at enrollment (kg/m^2^)^†^ | 23.6 (18.8-35.6) | 23.6 (18.8-35.6) |
| *Missing* | *7.5 (391)* | *0 (0)* |
| Smoking during pregnancy (%) |  |  |
| No | 77.2 (3,633) | 77.0 (4,007) |
| Yes | 22.8 (1,076) | 23.0 (1,195) |
| *Missing* | *9.5 (493)* | *0 (0)* |
| Psychiatric symptoms during pregnancy^†^ | 0.13 (0-1.29) | 0.13 (0-1.33) |
| *Missing* | *19.0 (990)* | *0 (0)* |
| **Child characteristics** |  |  |
| Sex (%) |  |  |
| Male | 49.6 (2,579) | 49.6 (2,579) |
| Female | 50.4 (2,623) | 50.4 (2,623) |
| *Missing* | *0 (0)* | *0 (0)* |
| Gestational age at birth (weeks)^†^ | 40.1 (36.0-42.3) | 40.1 (36.0-42.3) |
| *Missing* | *0.2 (9)* | *0 (0)* |
| Birth weight (grams)* | 3,454 (550) | 3,454 (549) |
| *Missing* | *0.1 (5)* | *0 (0)* |
| Ethnic origin (%) |  |  |
| European | 72.8 (3,750) | 72.5 (3,770) |
| Non-European | 27.2 (1,400) | 27.5 (1,432) |
| *Missing* | *1.0 (52)* | *0 (0)* |
| Breastfed ever (%) |  |  |
| No | 7.8 (399) | 8.4 (439) |
| Yes | 92.2 (4,709) | 91.6 (4,763) |
| *Missing* | *1.8 (94)* | *0 (0)* |
| Breastfeeding duration (%) |  |  |
| Never | 9.0 (399) | 8.4 (439) |
| <6 months | 58.3 (2,574) | 56.7 (2,949) |
| ≥6 months | 32.7 (1,443) | 34.9 (1,814) |
| *Missing* | *15.1 (786)* | *0 (0)* |
| Ointment use for eczema at age 2 months (%) |  |  |
| No | 93.0 (3,336) | 92.4 (4,809) |
| Yes | 7.0 (252) | 7.6 (393) |
| *Missing* | *31.0 (1,614)* | *0 (0)* |
| Cow's milk allergy until age 1 year (%) |  |  |
| No | 94.2 (4,856) | 94.1 (4,897) |
| Yes | 5.8 (301) | 5.9 (305) |
| *Missing* | *0.9 (45)* | *0 (0)* |
| Day care attendance until age 1 year (%) |  |  |
| No | 41.0 (1,767) | 42.5 (2,210) |
| Yes | 59.0 (2,541) | 57.5 (2,992) |
| *Missing* | *17.2 (894)* | *0 (0)* |
| Antibiotic use until age 1 year (%) |  |  |
| No | 77.8 (3,032) | 78.0 (4,059) |
| Yes | 22.2 (864) | 22.0 (1,143) |
| *Missing* | *25.1 (1,306)* | *0 (0)* |
| Body mass index at age 10-13 months (kg/m^2^)^†^ | 17.3 (14.9-20.3) | 17.3 (14.9-20.3) |
| *Missing* | *18.3 (954)* | *0 (0)* |
| Introduction of cow's milk (%) |  |  |
| ≤6 months | 73.9 (3,830) | 74.0 (3,847) |
| >6 months | 26.1 (1,351) | 26.0 (1,355) |
| *Missing* | *0.4 (21)* | *0 (0)* |
| Introduction of hen's egg (%) |  |  |
| ≤6 months | 11.5 (551) | 14.2 (741) |
| >6 months | 88.5 (4,244) | 85.8 (4,461) |
| *Missing* | *7.8 (407)* | *0 (0)* |
| Introduction of peanut (%) |  |  |
| ≤6 months | 2.9 (141) | 5.8 (303) |
| >6 months | 97.1 (4,647) | 94.2 (4,899) |
| *Missing* | *8.0 (414)* | *0 (0)* |
| Introduction of tree nuts (%) |  |  |
| ≤6 months | 0.5 (26) | 4.5 (236) |
| >6 months | 99.5 (4,717) | 95.5 (4,966) |
| *Missing* | *8.8 (459)* | *0 (0)* |
| Introduction of soy (%) |  |  |
| ≤6 months | 18.7 (930) | 20.3 (1,055) |
| >6 months | 81.3 (4,046) | 79.7 (4,147) |
| *Missing* | *4.3 (226)* | *0 (0)* |
| Introduction of gluten (%) |  |  |
| ≤6 months | 43.4 (2,196) | 44.2 (2,298) |
| >6 months | 56.6 (2,861) | 55.8 (2,904) |
| *Missing* | *2.8 (145)* | *0 (0)* |
| Diversity of allergenic foods introduced at age ≤6 months (%) |  |  |
| No allergenic foods introduced | 19.7 (916) | 18.2 (945) |
| 1 allergenic food introduced | 36.6 (1,705) | 33.7 (1,754) |
| 2 allergenic foods introduced | 31.0 (1,442) | 29.1 (1,516) |
| ≥3 allergenic foods introduced | 12.7 (594) | 19.0 (987) |
| *Missing* | *10.5 (545)* | *0 (0)* |
| Allergic sensitization at age 10 years – inhalant (%) |  |  |
| No | 68.5 (2,068) | 68.5 (2,068) |
| Yes | 31.5 (949) | 31.5 (949) |
| *Missing* | *42.0 (2,185)* | *42.0 (2,185)* |
| Allergic sensitization at age 10 years – food (%) |  |  |
| No | 93.3 (2,804) | 93.3 (2,804) |
| Yes | 6.7 (202) | 6.7 (202) |
| *Missing* | *42.2 (2,196)* | *42.2 (2,196)* |
| Physician-diagnosed allergy at age 10 years – inhalant (%) |  |  |
| No | 88.0 (3,182) | 88.0 (3,182) |
| Yes | 12.0 (435) | 12.0 (435) |
| *Missing* | *30.5 (1,585)* | *30.5 (1,585)* |
| Physician-diagnosed allergy at age 10 years – food (%) |  |  |
| No | 97.7 (3,465) | 97.7 (3,465) |
| Yes | 2.3 (81) | 2.3 (81) |
| *Missing* | *31.8 (1,656)* | *31.8 (1,656)* |
| Allergic sensitization and allergy combined at age 10 years (%) |  |  |
| No allergic sensitization and no allergy | 67.1 (1,759) | 67.1 (1,759) |
| Any allergic sensitization, but no allergy | 21.9 (574) | 21.9 (574) |
| No allergic sensitization, but any allergy | 1.2 (31) | 1.2 (31) |
| Any allergic sensitization and any allergy | 9.8 (258) | 9.8 (258) |
| *Missing* | *49.6 (2,580)* | *49.6 (2,580)* |
| Eczema last 6 months at age 6 months (%) |  |  |
| No | 83.8 (3,166) | 83.2 (4,374) |
| Yes | 16.2 (610) | 17.8 (928) |
| *Missing* | *27.4 (1,426)* | *0 (0)* |
| Eczema last 6 months at age 1 year (%) |  |  |
| No | 87.2 (4,009) | 86.7 (4,512) |
| Yes | 12.8 (589) | 13.3 (690) |
| *Missing* | *11.6 (604)* | *0 (0)* |
| Eczema last 12 months at age 2 years (%) |  |  |
| No | 86.7 (3,800) | 86.3 (4,490) |
| Yes | 13.3 (582) | 13.7 (712) |
| *Missing* | *15.8 (820)* | *0 (0)* |
| Eczema last 12 months at age 3 years (%) |  |  |
| No | 90.7 (3,701) | 90.0 (4,683) |
| Yes | 9.3 (380) | 10.0 (519) |
| *Missing* | *21.5 (1,121)* | *0 (0)* |
| Eczema last 12 months at age 4 years (%) |  |  |
| No | 92.2 (3,700) | 91.6 (4,762) |
| Yes | 7.8 (312) | 8.5 (440) |
| *Missing* | *22.9 (1,190)* | *0 (0)* |
| Eczema last 12 months at age 10 years (%) |  |  |
| No | 93.4 (3,447) | 90.1 (4,754) |
| Yes | 6.6 (245) | 8.6 (448) |
| *Missing* | *29.0 (1,510)* | *0 (0)* |

Values are *means (SD), ^†^medians (2.5-97.5th percentile) or percentages (absolute numbers) based on observed and imputed data. Data on allergic sensitizations and physician-diagnosed allergies are not imputed.
