# Supplementary material for: Allergenic food introduction and risk of childhood atopic diseases
Source: PLoS One. 2017 Nov 27;12(11):e0187999. doi: 10.1371/journal.pone.0187999 (PMC5703454; doi:10.1371/journal.pone.0187999)
Supplement: S3 Table — Values are *means (SD), †medians (2.5–97.5th percentile) or percentages (absolute numbers) based on observed data. P-values for difference are calculated by independent samples T-test for continuous variables with a normal distribution, the Mann-Whitney U test for continuous variables with a skewed distribution, and Pearson's Chi-square test for categorical variables. Bold values indicate statistical significance at the α = 0.05 level. (DOCX) [file pone.0187999.s004.docx]

**S3 Table. Characteristics of mothers and children included and not included in the study.**

|  | **Included**  **n = 5,202** | **Not included**  **n = 3,242** | **P-value for difference** |
| --- | --- | --- | --- |
| **Maternal characteristics** |  |  |  |
| Age at enrollment (years)* | 31.1 (4.8) | 28.8 (5.7) | **<0.001** |
| *Missing* | *0 (0)* | *0.1 (2)* |  |
| Education (%) |  |  | **<0.001** |
| Primary or secondary | 44.8 (2,229) | 75.0 (1,942) |  |
| Higher | 55.2 (2,751) | 25.0 (649) |  |
| *Missing* | *4.3 (222)* | *20.1 (651)* |  |
| History of allergy, eczema or asthma (%) |  |  | 0.68 |
| No | 60.9 (2,658) | 61.4 (1,401) |  |
| Yes | 39.1 (1,705) | 38.6 (879) |  |
| *Missing* | *16.1 (839)* | *29.7 (962)* |  |
| Parity (%) |  |  | **<0.001** |
| 0 | 58.7 (2,984) | 50.0 (1,533) |  |
| ≥1 | 41.3 (2,102) | 50.0 (1,535) |  |
| *Missing* | *2.2 (116)* | *5.4 (174)* |  |
| Pet keeping during pregnancy (%) |  |  | **<0.01** |
| No | 65.4 (2,774) | 69.4 (1,550) |  |
| Yes | 34.6 (1,467) | 30.6 (683) |  |
| *Missing* | *18.5 (961)* | *31.1 (1,009)* |  |
| Body mass index at enrollment (kg/m^2^)^†^ | 23.6 (18.8-35.6) | 24.4 (18.6-37.3) | **<0.001** |
| *Missing* | *7.5 (391)* | *14.5 (469)* |  |
| Smoking during pregnancy (%) |  |  | **<0.001** |
| No | 77.2 (3,633) | 68.1 (1,735) |  |
| Yes | 22.8 (1,076) | 31.9 (811) |  |
| *Missing* | *9.5 (493)* | *21.5 (696)* |  |
| Psychiatric symptoms during pregnancy^†^ | 0.13 (0-1.29) | 0.23 (0-1.64) | **<0.001** |
| *Missing* | *19.0 (990)* | *45.5 (1,474)* |  |
| **Child characteristics** |  |  |  |
| Sex (%) |  |  | **<0.05** |
| Male | 49.6 (2,579) | 51.9 (1,683) |  |
| Female | 50.4 (2,623) | 48.1 (1,557) |  |
| *Missing* | *0 (0)* | *0.1 (2)* |  |
| Gestational age at birth (weeks)^†^ | 40.1 (36.0-42.3) | 39.9 (34.1-42.2) | **<0.001** |
| *Missing* | *0.2 (9)* | *1.9 (62)* |  |
| Birth weight (grams)* | 3,454 (550) | 3,310 (608) | **<0.001** |
| *Missing* | *0.1 (5)* | *0.8 (27)* |  |
| Ethnic origin (%) |  |  | **<0.001** |
| European | 72.8 (3,750) | 47.9 (1,404) |  |
| Non-European | 27.2 (1,400) | 52.1 (1,529) |  |
| *Missing* | *1.0 (52)* | *9.5 (309)* |  |
| Breastfed ever (%) |  |  | **<0.05** |
| No | 7.8 (399) | 10.1 (118) |  |
| Yes | 92.2 (4,709) | 89.9 (1,050) |  |
| *Missing* | *1.8 (94)* | *64.0 (2,074)* |  |
| Day care attendance until age 1 year (%) |  |  | 0.52 |
| No | 41.0 (1,767) | 44.0 (51) |  |
| Yes | 59.0 (2,541) | 56.0 (65) |  |
| *Missing* | *17.2 (894)* | *96.4 (3,126)* |  |
| Antibiotic use until age 1 year (%) |  |  | 0.26 |
| No | 77.8 (3,032) | 73.6 (92) |  |
| Yes | 22.2 (864) | 26.4 (33) |  |
| *Missing* | *25.1 (1,306)* | *96.1 (3,117)* |  |
| Body mass index at age 10-13 months (kg/m^2^)^†^ | 17.3 (14.9-20.3) | 17.4 (14.7-20.6) | 0.29 |
| *Missing* | *18.3 (954)* | *63.5 (2,060)* |  |
| Introduction of cow's milk (%) |  |  | 0.32 |
| ≤6 months | 73.9 (3,830) | 70.2 (99) |  |
| >6 months | 26.1 (1,351) | 29.8 (42) |  |
| *Missing* | *0.4 (21)* | *95.7 (3,101)* |  |
| Introduction of hen's egg (%) |  |  | 0.09 |
| ≤6 months | 11.5 (551) | 6.6 (8) |  |
| >6 months | 88.5 (4,244) | 93.4 (114) |  |
| *Missing* | *7.8 (407)* | *96.2 (3,120)* |  |
| Introduction of peanut (%) |  |  | 0.83 |
| ≤6 months | 2.9 (141) | 3.3 (4) |  |
| >6 months | 97.1 (4,647) | 96.7 (118) |  |
| *Missing* | *8.0 (414)* | *96.2 (3,120)* |  |
| Introduction of tree nuts (%) |  |  | 0.70 |
| ≤6 months | 0.5 (26) | 0.8 (1) |  |
| >6 months | 99.5 (4,717) | 99.2 (122) |  |
| *Missing* | *8.8 (459)* | *96.2 (3,119)* |  |
| Introduction of soy (%) |  |  | 0.41 |
| ≤6 months | 18.7 (930) | 21.5 (29) |  |
| >6 months | 81.3 (4,046) | 78.5 (106) |  |
| *Missing* | *4.3 (226)* | *95.8 (3,107)* |  |
| Introduction of gluten (%) |  |  | **<0.05** |
| ≤6 months | 43.4 (2,196) | 32.6 (42) |  |
| >6 months | 56.6 (2,861) | 67.4 (87) |  |
| *Missing* | *2.8 (145)* | *96.0 (3,113)* |  |
| Diversity of allergenic foods introduction at age ≤6 months (%) |  |  | 0.09 |
| No allergenic foods introduced | 19.7 (916) | 28.3 (34) |  |
| 1 allergenic food introduced | 36.6 (1,705) | 37.5 (45) |  |
| 2 allergenic foods introduced | 31.0 (1,442) | 22.5 (27) |  |
| ≥3 allergenic foods introduced | 12.7 (594) | 11.7 (14) |  |
| *Missing* | *10.5 (545)* | *96.3 (3,122)* |  |

Values are *means (SD), ^†^medians (2.5-97.5th percentile) or percentages (absolute numbers) based on observed data. P-values for difference are calculated by independent samples T-test for continuous variables with a normal distribution, the Mann-Whitney U test for continuous variables with a skewed distribution, and Pearson's Chi-square test for categorical variables. Bold values indicate statistical significance at the α = 0.05 level.
