# Supplementary material for: Allergenic food introduction and risk of childhood atopic diseases
Source: PLoS One. 2017 Nov 27;12(11):e0187999. doi: 10.1371/journal.pone.0187999 (PMC5703454; doi:10.1371/journal.pone.0187999)
Supplement: S4 Table — Values are odds ratios (95% confidence interval) from multinomial logistic regression models based on imputed data. Reference group is children without any allergic sensitization or physician-diagnosed allergy (n = 1,759), and with *allergenic food introduction at age >6 months or †no allergenic foods introduced at age ≤6 months. Models are adjusted for maternal age at enrollment, education, history of allergy, eczema or asthma, parity, pet keeping, body mass index at enrollment, smoking, psychiatric symptoms, and child's sex, gestational age, birth weight, ethnic origin, breastfeeding, day care attendance and antibiotic use. N.A. = not available due to low number of children. (DOCX) [file pone.0187999.s005.docx]

**S4 Table. Associations of timing and diversity of allergenic food introduction with combined allergic sensitization and physician-diagnosed allergy groups in children at age 10 years.**

|  | **Odds ratio (95% confidence interval) for any allergic sensitization and any physician-diagnosed allergy**  **combined** | | |
| --- | --- | --- | --- |
|  | **Any allergic sensitization, but no allergy**  **n = 574** | **No allergic sensitization, but any allergy**  **n = 31** | **Any allergic sensitization and any allergy**  **n = 258** |
| Allergenic food introduced at age ≤6 months* |  |  |  |
| Cow's milk (n = 3,847) | 0.89 (0.71, 1.12) | 0.55 (0.24, 1.24) | 0.84 (0.61, 1.14) |
| Hen's egg (n = 741) | 1.12 (0.82, 1.52) | 0.48 (0.11, 2.17) | 0.86 (0.52, 1.43) |
| Peanut (n = 303) | 0.67 (0.36, 1.25) | N.A. | 0.83 (0.39, 1.78) |
| Tree nuts (n = 236) | 1.04 (0.46, 2.33) | N.A. | 1.08 (0.26, 4.49) |
| Soy (n = 1,055) | 1.00 (0.77, 1.28) | 1.24 (0.50, 3.05) | 1.15 (0.82, 1.60) |
| Gluten (n = 2,904) | 0.93 (0.76, 1.14) | 0.45 (0.20, 1.02) | 0.95 (0.72, 1.27) |
| Diversity of allergenic foods introduced at age ≤6 months^†^ |  |  |  |
| 1 allergenic food introduced (n = 1,754) | 0.84 (0.63, 1.12) | 0.54 (0.20, 1.47) | 0.88 (0.59, 1.32) |
| 2 allergenic foods introduced (n = 1,516) | 0.82 (0.61, 1.10) | 0.48 (0.17, 1.37) | 0.89 (0.59, 1.35) |
| ≥3 allergenic foods introduced (n = 987) | 0.88 (0.62, 1.23) | 0.30 (0.08, 1.12) | 0.88 (0.54, 1.43) |
| P-value for trend | 0.27 | 0.55 | 0.63 |

Values are odds ratios (95% confidence interval) from multinomial logistic regression models based on imputed data. Reference group is children without any allergic sensitization or physician-diagnosed allergy (n = 1,759), and with *allergenic food introduction at age >6 months or ^†^no allergenic foods introduced at age ≤6 months. Models are adjusted for maternal age at enrollment, education, history of allergy, eczema or asthma, parity, pet keeping, body mass index at enrollment, smoking, psychiatric symptoms, and child's sex, gestational age, birth weight, ethnic origin, breastfeeding, day care attendance and antibiotic use. N.A. = not available due to low number of children.
