# Supplementary material for: Allergenic food introduction and risk of childhood atopic diseases
Source: PLoS One. 2017 Nov 27;12(11):e0187999. doi: 10.1371/journal.pone.0187999 (PMC5703454; doi:10.1371/journal.pone.0187999)
Supplement: S5 Table — Values are odds ratios (95% confidence interval) from generalized estimating equation models based on imputed data. Bold values indicate statistical significance at the α = 0.05 level. Reference group is children without any allergic sensitization or physician-diagnosed allergy, and with *allergenic food introduction at age >6 months or †no allergenic foods introduced at age ≤6 months. Models are adjusted for maternal age at enrollment, education, history of allergy, eczema or asthma, parity, pet keeping, body mass index at enrollment, smoking, psychiatric symptoms, and child's sex, gestational age, birth weight, ethnic origin, breastfeeding, day care attendance and antibiotic use. (DOCX) [file pone.0187999.s006.docx]

**S5 Table. Associations of timing and diversity of allergenic food introduction with eczema per year and overall in children until age 10 years.**

|  | **Odds ratio (95% confidence interval) for eczema** | | | | | | |
| --- | --- | --- | --- | --- | --- | --- | --- |
|  | **6 months** | **1 year** | **2 years** | **3 years** | **4 years** | **10 years** | **Overall** |
| Allergenic food introduced at age ≤6 months* |  |  |  |  |  |  |  |
| Cow's milk (n = 3,847) | 0.62 (0.32, 1.22) | 0.86 (0.71, 1.06) | 0.85 (0.70, 1.04) | 0.93 (0.74, 1.17) | 1.12 (0.85, 1.48) | 1.01 (0.78, 1.33) | 0.82 (0.63, 1.07) |
| Hen's egg (n = 741) | 0.74 (0.54, 1.01) | 0.67 (0.42, 1.07) | 0.79 (0.58, 1.09) | 0.80 (0.53, 1.22) | 1.06 (0.73, 1.54) | 1.27 (0.85, 1.88) | 0.87 (0.73, 1.03) |
| Peanut (n = 303) | 0.68 (0.38, 1.21) | 0.79 (0.30, 2.13) | 0.80 (0.45, 1.41) | 0.77 (0.38, 1.57) | 0.67 (0.28, 1.59) | 0.89 (0.39, 2.02) | 0.77 (0.49, 1.22) |
| Tree nuts (n = 236) | 0.89 (0.30, 2.61) | 1.07 (0.36, 3.15) | 0.93 (0.46, 1.88) | 1.10 (0.40, 2.97) | 1.21 (0.47, 3.11) | 1.25 (0.49, 3.20) | 1.08 (0.61, 1.91) |
| Soy (n = 1,055) | 0.88 (0.68, 1.13) | 0.98 (0.70, 1.36) | 0.97 (0.75, 1.24) | 0.98 (0.73, 1.32) | 1.20 (0.88, 1.62) | 1.15 (0.84, 1.57) | 1.00 (0.87, 1.15) |
| Gluten (n = 2,904) | 0.71 (0.50, 1.01) | **0.75 (0.60, 0.95)** | 0.88 (0.73, 1.06) | 0.93 (0.73, 1.18) | 0.91 (0.72, 1.17) | 1.07 (0.80, 1.44) | **0.84 (0.72, 0.99)** |
| Diversity of allergenic foods introduced at age ≤6 months^†^ |  |  |  |  |  |  |  |
| 1 allergenic food  introduced (n = 1,754) | 0.75 (0.41, 1.36) | 0.99 (0.79, 1.25) | 0.88 (0.69, 1.12) | 0.98 (0.73, 1.31) | 0.98 (0.71, 1.36) | 1.02 (0.72, 1.45) | 0.88 (0.69, 1.13) |
| 2 allergenic foods introduced (n = 1,516) | 0.58 (0.29, 1.19) | 0.81 (0.64, 1.04) | 0.90 (0.70, 1.15) | 0.95 (0.70, 1.29) | 1.10 (0.79, 1.52) | 1.07 (0.75, 1.53) | 0.82 (0.60, 1.11) |
| ≥3 allergenic foods introduced (n = 987) | 0.52 (0.24, 1.13) | 0.67 (0.43, 1.06) | **0.71 (0.51, 0.99)** | 0.84 (0.56, 1.26) | 1.07 (0.71, 1.60) | 1.17 (0.75, 1.85) | 0.75 (0.54, 1.03) |
| P-value for trend | 0.52 | 0.51 | 0.77 | 0.89 | 0.80 | 0.78 | 0.57 |

Values are odds ratios (95% confidence interval) from generalized estimating equation models based on imputed data. Bold values indicate statistical significance at the α = 0.05 level. Reference group is children without any allergic sensitization or physician-diagnosed allergy, and with *allergenic food introduction at age >6 months or ^†^no allergenic foods introduced at age ≤6 months. Models are adjusted for maternal age at enrollment, education, history of allergy, eczema or asthma, parity, pet keeping, body mass index at enrollment, smoking, psychiatric symptoms, and child's sex, gestational age, birth weight, ethnic origin, breastfeeding, day care attendance and antibiotic use.
